# Aortic Dissection and Thrombosis Diagnosed by Emergency Ultrasound in a Patient with Leg Pain and Paralysis

**DOI:** 10.1155/2013/490126

**Published:** 2013-01-31

**Authors:** Ann H. Tsung, Leslie C. Nickels, Giuliano De Portu, Eike F. Flach, Latha Ganti Stead

**Affiliations:** Department of Emergency Medicine, College of Medicine, University of Florida, 1329-SW 16 Street, P.O. Box 100186, Gainesville, FL 32610, USA

## Abstract

The authors present a case of aortic dissection and abdominal aortic aneurysm thrombosis in a 78-year-old male who presented to the emergency department (ED) complaining of lower extremity and paralysis for the past 1.5 hours. The initial vital signs in the ED were as follows: blood pressure (BP) 132/88 mmHg, heart rate (HR) 96, respiratory rate (RR) 14, and an oxygen saturation of 94% at room air. Physical exam was notable for pale and cold left leg. The ED physician was unable to palpate or detect a Doppler signal in the left femoral artery. Bedside ultrasound was performed which showed non-pulsatile left femoral artery and limited flow on color Doppler. Abdominal aortic aneurysm screening ultrasound was performed showing a 4.99 cm infrarenal abdominal aortic aneurysm and an intra-aortic thrombus with an intimal flap. Vascular surgery was promptly contacted and the patient underwent emergent aorto-bi-femoral bypass, bilateral four compartment fasciotomy, right common femoral artery endarterectomy with profundoplasty, and subsequent left leg amputation. Emergency physicians should utilize bedside ultrasound in patients who present with risk factors or threatening signs and symptoms that may suggest aortic dissection or aneurysm. Bedside ultrasound decreases time to definitive treatment and the mortality of the patients.

## 1. Introduction

Aortic aneurysm often goes undiagnosed and carries an extremely high mortality rate when ruptured. Here, we present a case of aortic dissection and complete infrarenal abdominal aortic aneurysm (AAA) thrombosis diagnosed by bedside ultrasound in the ED. 

## 2. Case Report

A 78-year-old Caucasian male with past medical history of chronic obstructive pulmonary disease, coronary artery disease, and peripheral vascular disease presented to the ED via helicopter for left lower extremity paralysis and a “cold extremity” that started an hour and half ago. The patient reported that he had some vague pain shortly before but now the pain was sharp and rated as 10 out of 10 on the pain scale. The patient was able to walk to the bathroom without aid this morning and is usually able to ambulate half a block before getting tired. The patient denied fever, loss of consciousness, nausea, vomiting, neck stiffness, photophobia, or aura. His home medications included acetaminophen and hydrocodone. The patient had no allergies. Prior surgeries included coronary artery bypass graft and two stents. The patient had a 64 pack year smoking history and denied alcohol or drug use.

**Figure 1:**
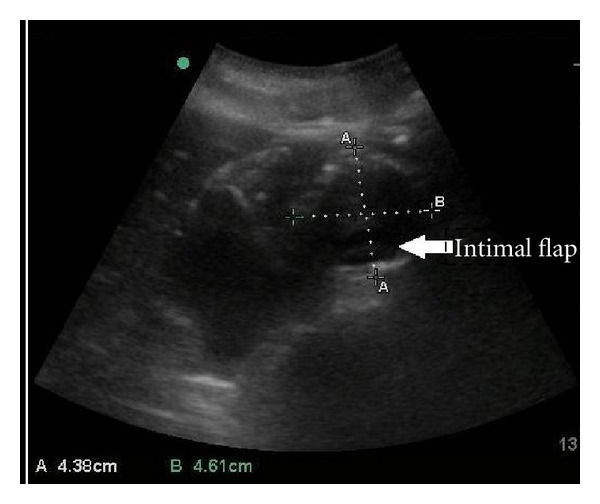
Abdominal ultrasonography demonstrating intimal flap (arrow).

**Figure 2:**
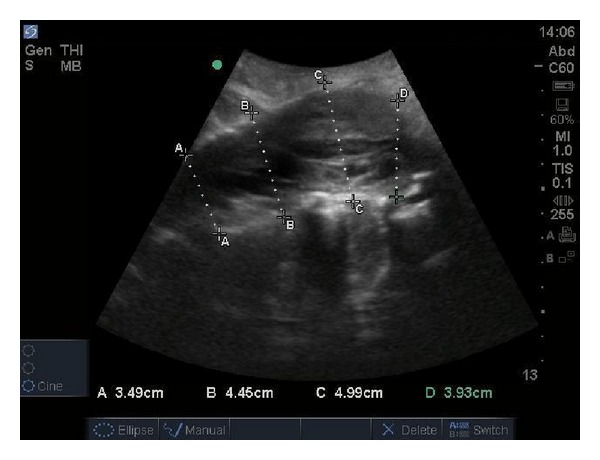
Abdominal ultrasonography demonstrating width measurements of aorta.

The patient's initial vital signs in the ED were as follows: blood pressure (BP) 132/88 mmHg, heart rate (HR) 96, respiratory rate (RR) 14, and an oxygen saturation of 94% at room air. He was alert and oriented without acute distress. Heart sounds were regular, with no murmur, click, bruit, or rubs noted. Breath sounds were clear to auscultation bilaterally. His abdomen was soft, nontender, with no pulsatile mass, rebound, or guarding. The left lower extremity was pale and cold compared to the contralateral leg with strength at a one out of five. The ED physician was unable to palpate or detect a Doppler signal in the left femoral, popliteal, posterior tibialis, and dorsalis pedis arteries. Strength was 5/5 in right lower extremity. No edema or erythema was present. Bedside ultrasound was performed which showed nonpulsatile bilateral femoral artery and limited flow. The decision was made to perform an AAA screening ultrasound, which showed a 4.61 cm infrarenal AAA and an intimal flap (Figure 1). The sagittal view showed a 4.99 cm AAA and complete aortoocclusion by the thrombus (Figure 2).

Emergent CT-angiography (CTA) was performed, which showed a thrombosed 5.5 cm infrarenal aortic aneurysm. The dissection was not detected since the contrast was unable to pass through the thrombus. Vascular surgery was promptly contacted and the patient underwent emergent aortobifemoral bypass, bilateral four-compartment fasciotomy, right common femoral artery endarterectomy with profundoplasty, and subsequent left above the knee amputation. 

## 3. Discussion

This was a case of aortic dissection and thrombosed AAA detected by bedside ultrasound in the ED with an initial presentation of lower extremity pain and paralysis. The incidence of AAA is 2–5% in men over age 50 and 10% in men with risk factors [1]. They include hypertension, smoking, atherosclerotic disease, peripheral vascular disease, stroke, diabetes, and family history of AAA [2]. It is 4 times more prevalent in men than women The normal diameter of the abdominal aorta is less than 3 cm. The risk of rupture per year with aortic diameter of 4 cm or less is less than 2%, 4-5 cm is 1–5%, 5-6 cm is 3–15%, 6-7 cm is 10–20%, and over 7 cm is 20–50%. Operative repair should start at 5.5 cm since the mortality of rupture is 80%. 40–50% of patients will die despite emergent surgery [1].

The classic triad of AAA is abdominal, back, or flank pain, hypotension, and pulsatile mass, which is present in 80%, 50%, and 18% of the AAA patients, respectively. Since less than 25% of the patients presents with all three, ED physicians should consider screening ultrasounds in patients who present with pain in the abdomen, back, flank, or groin, dizziness, syncope, unexplained hypotension, or cardiac arrest. It has been shown that bedside ultrasound is 100% sensitive and 98–100% specific in detecting AAA [1]. Bedside ultrasound has also been shown to decrease time to diagnosis from 83 minutes to 5.4 minutes and disposition for surgery from 90 minutes to 12 minutes. Most importantly, it decreases mortality from 72% to 40% [1]. In this case, vascular surgery was contacted 30 minutes after the patient arrived to the ED. When radiology contacted the ED physician on the critical read of the CTA, the patient was already transferred for operative repair. 

There is only one other case report that described a complete AAA thrombosis in a patient that presented with severe bilateral lower extremity pain, weakness, and numbness, which is similar to the patient in this case. The incidence of complete acute thrombosis of AAA is 0.7–2.8% with a mortality rate of 46% [3]. Classical signs of aortic thrombosis on ultrasound include absence of arterial pulsations, vessel lumen with echogenic material, and absence of flow on Doppler. The patient in this case had echogenic material in the aorta, absence of flow, and absent aortic pulsations distal to the obstruction. 

It is interesting to note that the aortic dissection in this case was not detected on the CTA due to absence of contrast flow through the aortic thrombus. Bedside ultrasound is actually superior to CTA in this particular circumstance. In general, use of contrast-enhanced ultrasound (CEUS) is comparable CTA for detection of aortic dissections with a sensitivity of 97% [4]. CEUS can also detect aortic ruptures, differentiation of true and false lumen, flow direction within the true and false lumen, aneurysms, and endoleaks after endovascular repair. The patient is this case did not use CEUS, but this modality can be considered for patients who are too unstable for CTA, allergic to contrast, or have an aortic thrombus. 

The other type of dissection is intrathoracic which requires a high clinical index of suspicion. The sign of mediastinum widening on chest radiograph is only 67% sensitive [5]. In comparison, the transthoracic echocardiogram (TTE) is 94–100% sensitive and 77–100% specific in detecting an intimal flap for aortic dissection [5]. Two echocardiogram features suggest thoracic aortic dissection. One is dilation of any segment of thoracic aorta with a 95% sensitivity and 51% specificity. Second is visualization of intimal flap with a 67% sensitivity and 100% specificity. TTE also has a positive predictive value of 97.5%, which is comparable to the 100% positive predictive value for CT. The TTE measurement of the thoracic aortic diameter has been shown to be in agreement with CTA with a mean difference between 1.5 and 2.2 mm [6]. 

Even though the TTE is a sensitive test for thoracic aortic dissection, the parasternal view is not used often while assessing for aortic dissection. The subxiphoid view may be an option for the emergency physicians. There have been seven papers that suggest that the presence of prominent Mercedes Benz sign of the aortic valve in subxiphoid view could indicate proximal aortic dissection [7]. Since the typical aortic root diameter is less than 3.8 cm and dilated root from a dissection can be over 5.0 cm, it changes the anatomic relationships in a small area. This alters the window provided for ultrasound beam transmission and the view obtained. Also the dissection to the aortic valve itself may change the perceived orientation and allow the ultrasound beam to deliver images from an angle that was not previously possible.

## 4. Conclusion

 Emergency physicians should perform screening bedside ultrasounds for aortic aneurysm and dissection if a patient has risk factors including hypertension, smoking, atherosclerotic disease, peripheral vascular disease, stroke, diabetes, and family history of AAA. One should also consider ultrasound in patients who present with pain in abdomen, back, flank, or groin, dizziness, syncope, unexplained hypotension, cardiac arrest, or limb ischemia. The abdominal aortic ultrasound, TTE, and CEUS are comparable to CT angiogram in detecting aortic aneurysm and aortic dissection. Since the TTE is not often performed when screening for AAA, a prominent Mercedes Benz sign in the subxiphoid view may indicate a thoracic aortic dissection. Aortic thrombosis should be considered when a patient presents with hemiplegia or paraplegia with neurologic deficits. Most importantly, the use of bedside ultrasound decreases time to definitive treatment and the mortality of the patients.
